# An *In Silico* Cardiomyocyte Reveals the Impact of Changes in CaMKII Signalling on Cardiomyocyte Contraction Kinetics in Hypertrophic Cardiomyopathy

**DOI:** 10.1155/2024/6160554

**Published:** 2024-03-25

**Authors:** Ismail Adeniran, Hafsa Wadee, Hans Degens

**Affiliations:** ^1^Centre for Advanced Computational Science, Manchester Metropolitan University, Manchester M15 6BH, UK; ^2^Department of Life Sciences, Manchester Metropolitan University, Manchester M15 6BH, UK; ^3^Lithuanian Sports University, Sporto 6, LT-44221 Kaunas, Lithuania

## Abstract

Hypertrophic cardiomyopathy (HCM) is characterised by asymmetric left ventricular hypertrophy, ventricular arrhythmias, and cardiomyocyte dysfunction that may cause sudden death. HCM is associated with mutations in sarcomeric proteins and is usually transmitted as an autosomal-dominant trait. The aim of this *in silico* study was to assess the mechanisms that underlie the altered electrophysiological activity, contractility, regulation of energy metabolism, and crossbridge cycling in HCM at the single-cell level. To investigate this, we developed a human ventricular cardiomyocyte model that incorporates electrophysiology, metabolism, and force generation. The model was validated by its ability to reproduce the experimentally observed kinetic properties of human HCM induced by (a) remodelling of several ion channels and Ca^2+^-handling proteins arising from altered Ca^2+^/calmodulin kinase II signalling pathways and (b) increased Ca^2+^ sensitivity of the myofilament proteins. Our simulation showed a decreased phosphocreatine-to-ATP ratio (-9%) suggesting a negative mismatch between energy expenditure and supply. Using a spatial myofilament half-sarcomere model, we also compared the fraction of detached, weakly bound, and strongly bound crossbridges in the control and HCM conditions. Our simulations showed that HCM has more crossbridges in force-producing states than in the control condition. In conclusion, our model reveals that impaired crossbridge kinetics is accompanied by a negative mismatch between the ATP supply and demand ratio. This suggests that improving this ratio may reduce the incidence of sudden death in HCM.

## 1. Introduction

Hypertrophic cardiomyopathy (HCM) is the most common inherited cardiac disorder, affecting 1 in 500 individuals, and is clinically characterised by left ventricular (LV) hypertrophy, a decrease in the LV chamber size, heart failure, arrhythmias, and sudden cardiac death at any age [1–3]. While the ejection fraction (EF) is preserved, the rate of relaxation is diminished [3–5]. It usually results from mutations of sarcomeric proteins, the sarcomere being the fundamental contractile unit of the cardiomyocyte [1, 3]. Histopathological features include disorganized myocyte architecture, including disarray of myocyte fibres, intertwined hypertrophied myocytes, and focal or widespread interstitial fibrosis [6, 7].

Over the past two decades, it has been suggested that an energy deficit plays an essential role in the aetiology of HCM [1, 8–10]. Most mutations in HCM lead to an increase in both the total force production and adenosine triphosphate (ATP) utilisation at the cellular level, consequently leading to a greater ATP demand by the cardiomyocytes [9, 11]. Notwithstanding this increased demand for ATP, both murine models [12, 13] and patients [14] with HCM exhibit an impaired, rather than enhanced, mitochondrial function and morphology. Despite significant advances in elucidating sarcomeric structure-function relationships and evidence linking impaired energy metabolism with the aetiology of HCM, there is still much to be discovered about the mechanisms that link altered cardiac energetics to HCM phenotypes due to insufficient cell and animal models and, more importantly, human HCM patient studies [9, 15].

Studies indicate that the pathogenesis of HCM involves a broad range of mechanisms, the primary mechanism of which is sarcomeric protein mutation. Molecular changes that occur in response to the changes in the sarcomere protein structure and function form a secondary mechanism, with histological and pathological phenotypes as a consequence of the molecular perturbations being tertiary mechanisms [1, 5, 6, 9]. One such tertiary mechanism may be the complex remodelling of Ca^2+^/calmodulin-dependent protein kinase II- (CaMKII-) dependent signalling [16, 17].

Advancements in understanding HCM will be facilitated by comprehensive models that can integrate complex factors influencing cardiomyocyte function. Indeed, previous models have made valuable contributions to understanding electromechanical characteristics in HCM. Computational modelling of cardiomyocytes in HCM revealed that the electromechanical features in HCM include delayed afterdepolarisations, prolonged action potentials, Ca^2+^ overload, and hyperdynamic contraction, related to an elevation in Ca^2+^-related current densities and differences in Na^+^-, K^+^-, and Cl^−^-related current densities compared to normal conditions [18]. The model of Pioner et al. [19] found that electrophysiological changes (prolonged action potentials and Ca^2+^ transients) were responsible for a preserved twitch duration in a HCM-linked MYBPC3 mutation. Another model study saw in cardiomyocytes from HCM patients several abnormalities, including prolonged action potentials due to increased late sodium (*I*_NaL_) and calcium (*I*_CaL_) currents along with decreased repolarizing potassium currents, increased arrhythmias, abnormal calcium handling, and increased CaMKII activity and phosphorylation [17]. Our *in silico* study applies a human ventricular cardiomyocyte model to assess how functional changes in CaMKII-dependent signalling impact electrophysiological activity, contractility, and energy metabolism regulation at the level of the single cardiomyocyte in human HCM.

Our single-cell model offers several advantages for studying HCM. First, it integrates electrophysiology, metabolism, and force generation, providing a holistic understanding of the altered cellular dynamics in HCM. Second, our model incorporates experimentally observed kinetic properties of HCM involving ion channel remodelling and altered Ca^2+^ handling. This ensures that our simulations closely resemble the actual conditions in HCM. Third, the spatial myofilament half-sarcomere model allows us to explore the distribution of crossbridges in force-producing states, providing insights into the mechanical aspects of HCM at a mesoscopic level. The model includes explicit representations of actin and myosin filaments. Although it is not an atomic-scale model, the model seeks to represent spatial interactions between protein complexes that are thought to produce characteristic cardiac muscle responses at larger scales. Our work thus advances the HCM research by offering an encompassing approach to electrophysiology, metabolism, and force generation within a single-cell context.

## 2. Materials and Methods

### 2.1. Electromechanical, Mitochondrial Energetics Single-Cell Model

For electrophysiology (EP), we employed the O'Hara-Rudy (ORd) human ventricular single-cell model [20]. We selected the ORd model because it is based on experimental data from human hearts and validated using cellular behaviours and mechanisms from an extensive dataset including measurements from over 100 undiseased human hearts. It accurately reproduces the electrical and membrane channel characteristics of human ventricular cells, as well as the transmural heterogeneity of ventricular action potentials (AP) across the ventricular wall. Furthermore, the ORd model replicates the modulation of the rate of Ca^2+^ cycling by CaMK, which is particularly important in the context of this study. It also reproduces the Ca^2+^- vs. voltage-dependent inactivation of the L-type Ca^2+^ current.

To simulate cardiomyocyte contractile properties, we utilised the myofilament model developed by Tran et al. [21], an extension of the established Rice et al. crossbridge cycling model of cardiac muscle contraction [22]. Not only can this myofilament model replicate a variety of experimental data, such as steady-state force-sarcomere length (F-SL), force-Ca^2+^, and sarcomere length-Ca^2+^ relationships, but it can also reproduce many of the effects on force development due to MgATP, MgADP, Pi, and H^+^ [21, 22]. In addition to these advantages, in the context of this study, the Tran et al. [21] model allowed us to assess the Hill coefficient to reproduce the change in the F-Ca^2+^ relationship in HCM seen by Coppini et al. [16, 17].

#### 2.1.1. Coupling the Electrophysiology and the Myofilament Models

The electrophysiology (EP) model was coupled with the myofilament model to produce an electromechanical cell model. The intracellular calcium concentration [Ca^2+^]_*i*_ from the EP model served as the link between the EP model and the myofilament model. Specifically, the dynamic [Ca^2+^]_*i*_ generated by the EP model in response to an AP was used as input to the myofilament model, which then calculated the quantity of Ca^2+^ bound to troponin.

The myoplasmic [Ca^2+^]_*i*_ concentration in the human ventricular myocyte electromechanical cell model is formulated as follows:

(1) $$\begin{array}{c} \frac{d{\left[{\text{Ca}}^{2+}\right]}_{i}}{dt}=\beta_{\text{Ca}i}∙\left(-\left(I_{p\text{Ca}}+I_{\text{Ca}b}-2∙I_{\text{NaCa},i}\right)∙\frac{A_{\text{cap}}}{2∙F∙v_{\text{myo}}}-J_{\text{up}}∙\frac{v_{\text{nsr}}}{v_{\text{myo}}}+J_{\text{diff},\text{Ca}}∙\frac{v_{\text{ss}}}{v_{\text{myo}}}-\frac{J_{\text{Trop}}}{1000}\right), \end{array}$$

where *β*_Ca*i*_ is the buffer factor for [Ca^2+^]_*i*_, *I*_*p*Ca_ (*μ*A/*μ*F) is the sarcolemmal Ca^2+^ pump current, *I*_Ca*b*_ (*μ*A/*μ*F) is the Ca^2+^ background current, *I*_NaCa,*i*_ (*μ*A/*μ*F) is the myoplasmic component of Na^+^/Ca^2+^ exchange current, *A*_cap_ (cm^2^) is the capacitive area, *F* (Coulomb/mol) is the Faraday constant, *v*_myo_ (*μ*L) is the volume of the myoplasmic compartment, *v*_nsr_ (*μ*L) is the volume of the sarcoplasmic reticulum compartment, *v*_ss_ (*μ*L) is the volume of the subspace compartment, *J*_up_ (mM/ms) is the total Ca^2+^ uptake flux via the SERCA pump from the myoplasm to the sarcoplasmic reticulum, *J*_diff,Ca_ (mM/ms) is the diffusion flux of Ca^2+^ from the subspace to the myoplasm, and *J*_Trop_ (*μ*M/ms) is the flux of Ca^2+^ binding to troponin.

*β*_Ca*i*_ is formulated as follows:

(2) $$\begin{array}{c} \beta_{\text{Ca}i}=\frac{1}{1+\left(\left(\left[\text{CMDN}\right]∙K_{m,\text{CMDN}}\right)/{\left(K_{m,\text{CMDN}}+{\left[{\text{Ca}}^{2+}\right]}_{i}\right)}^{2}\right)}, \end{array}$$

where [CMDN] is the calmodulin Ca^2+^ buffer in the myoplasm and *K*_*m*,CMDN_ is the half-saturation concentration of calmodulin.

#### 2.1.2. Coupling the Electromechanical Model to the Mitochondrial Model

For cardiac mitochondrial bioenergetics, we then coupled this electromechanical single-cell model to the thermokinetic mitochondrial model of Cortassa et al. [23]. This model describes the tricarboxylic acid (TCA) cycle, oxidative phosphorylation, and mitochondrial Ca^2+^ handling. It can reproduce, qualitatively and semiquantitatively, experimental data concerning mitochondrial bioenergetics, Ca^2+^ dynamics, and respiratory control.

To consider mitochondrial energetics, modifications were made to the sarcolemmal Ca^2+^ pump current (*I*_*p*Ca_) and the SERCA pump uptake flux (*J*_up_) of the electromechanical single-cell model as follows:

(3) $$\begin{array}{c} I_{p\text{Ca}}=G_{p\text{Ca}}∙\frac{{\left[{\text{Ca}}^{2+}\right]}_{i}}{0.0005+{\left[{\text{Ca}}^{2+}\right]}_{i}}∙\frac{1}{1+\left(K_{m1\_p\text{Ca}}^{\text{ATP}}/{\left[\text{ATP}\right]}_{i}\right)∙\left(1+\left({\left[\text{ADP}\right]}_{i}/K_{i\_p\text{Ca}}^{\text{ADP}}\right)\right)}+\frac{1}{1+\left(K_{m2\_p\text{Ca}}^{\text{ATP}}/{\left[\text{ATP}\right]}_{i}\right)}, \end{array}$$

where *G*_*p*Ca_ is the maximum conductance of *I*_*p*Ca_, *K*_*m*1_*p*Ca_^ATP^ is the first ATP (adenosine triphosphate) half-saturation constant for *I*_*p*Ca_, *K*_*m*2_*p*Ca_^ATP^ is the second ATP half-saturation constant for *I*_*p*Ca_, *K*_*i*_*p*Ca_^ADP^ is the ADP inhibition constant for *I*_*p*Ca_, [ATP]_*I*_ is the excitation-contraction (EC) coupling-linked ATP concentration, and [ADP]_*I*_ is the excitation-contraction (EC) coupling-linked ADP (adenosine diphosphate) concentration.

(4) $$\begin{array}{c} J_{\text{up}}=\left(\left(1-\emptyset_{\text{up},\text{CaMK}}\right)∙J_{\text{up},NP}+\emptyset_{\text{up},\text{CaMK}}∙J_{\text{up},\text{CaMK}}-J_{\text{leak}}\right)∙\left(\frac{\left(K_{\text{SR}}^{m\text{ATP}}+{\left[\text{ADP}\right]}_{i}∙K_{\text{SR}\_\text{Ki}\_\text{SR}}^{m\text{ATP}}\right)}{{\left[\text{ATP}\right]}_{i}}+\left(1+\frac{{\left[\text{ADP}\right]}_{i}}{{\text{Ki}}_{\text{SR}}^{\prime }}\right)\right), \end{array}$$

where *Ø*_up,CaMK_ is the fraction of channels phosphorylated by CaMK, *J*_up,NP_ is the nonphosphorylated Ca^2+^ uptake by CaMK via the SERCA pump, *J*_up,CaMK_ is the CaMK-phosphorylated Ca^2+^ uptake via the SERCA pump, and *J*_leak_ is the leakage flux.

In addition to the above, the main driver of the interaction between the electromechanical model and the mitochondrial model is the [Ca^2+^]_*i*_ from the electrophysiology model. In the mitochondrial model, it influences processes such as ATP production. The mitochondrial model then feeds back to the electromechanical model to influence Ca^2+^ cycling and Ca^2+^ handling.

### 2.2. HCM Electromechanical Single-Cell Model

To develop the human electromechanical single-cell model of a HCM cardiomyocyte, we modified the EP and myofilament models to reflect the experimental data from Coppini et al. [16, 17] on the electromechanical profile of cardiomyocytes from 26 HCM patients. We made the following changes to the electromechanical single-cell model (see Table 1).

### 2.3. Spatially Explicit Multifilament Half-Sarcomere Model

**Figure 1:**
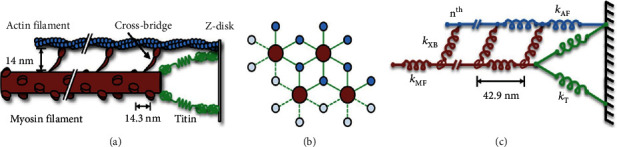
Spatially explicit half-sarcomere model (modified from [24]). (a) Schematic of the half-sarcomere showing the myosin filament (red), actin filament (blue), titin (green), Z-disk (black), and crossbridges (red). (b) Spatial hexagonal arrangement of myosin and actin filaments. (c) Mathematically, the half-sarcomere is modelled as an array of springs: myosin and crossbridges (red), actin (blue), and titin (green).

To further investigate the pathological behaviour in HCM, we also employed a spatially explicit multifilament computational model of the half-sarcomere [24–27]. This model is comprised of eight actin filaments and four myosin filaments arranged in a three-dimensional double-hexagonal lattice (see Figures 1(a) and 1(b)). It simulates an infinite lattice using periodic (toroidal) boundary conditions (see Figure 1(b)). Each crossbridge is modelled as a two-spring system consisting of a torsional and linear component. Linear springs are connected in series between each node of crossbridge crowns for the myosin and actin filaments (see Figure 1(c)). Each myosin filament contains 60 crowns of three myosin motors, resulting in 180 crossbridges per myosin filament.

Importantly, the model possesses two noteworthy characteristics that distinguish it from other existing models. First, it integrates torsional springs and lever-arm mechanisms that expose a correlation between step size and lattice spacing. Second, this lever-arm mechanism generates both radial and axial forces of similar magnitude, mirroring experimental findings [26].

**Figure 2:**
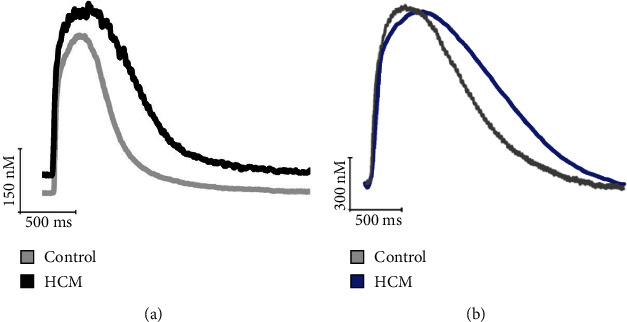
[Ca^2+^]_*i*_ transients recorded from control and HCM cardiomyocytes input into the multifilament half-sarcomere model. (a) Transients recorded in current clamp mode from a study on 26 HCM patients [17]. (b) Transients recorded from a study on 96 patients with a MYBPC3 mutation [19].

As input to the multifilament half-sarcomere model, we digitised [Ca^2+^]_*i*_ transients from control and HCM cardiomyocytes recorded in two separate studies. The first set comes from 26 HCM patients in [16, 17] (see Figure 2(a)). The second set is from 93 HCM patients with a myosin-binding protein C (MYBPC3) mutation [19]. Mutations to MYBPC3 are the most common cause of HCM [28].

**Figure 3:**
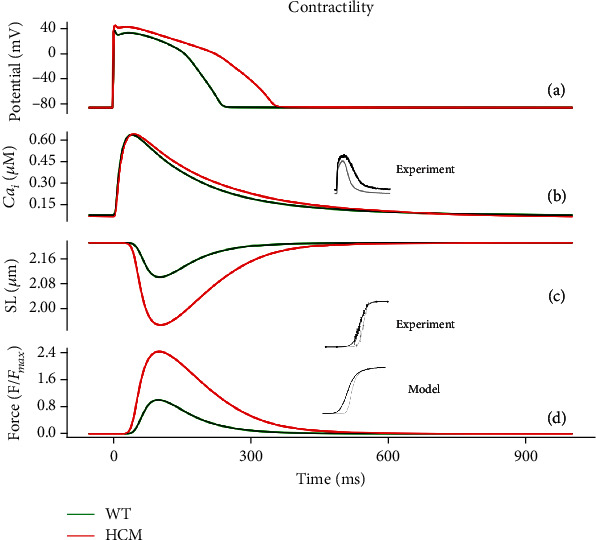
Contractile properties under control (green) and HCM (red) at 1 Hz cycle length. (a) Epicardial action potentials in HCM and control. (b) [Ca^2+^]_*i*_ transients. (c) Sarcomere length (SL). (d) Active force. Values are normalised to control the maximum active force.

In each case, the maximum [Ca^2+^]_*i*_ for the control condition was set equivalent to an actin permissiveness of 1 during crossbridge cycling while the minimum [Ca^2+^]_*i*_ for the control condition was set equivalent to an actin permissiveness of 0.2. Values of actin permissiveness were proportionally adjusted between these values during the time course simulation of the prerecorded profiles for both control and HCM conditions. Each timepoint of each calcium transient profile was run for 100 ms until reaching a steady state, at which point the steady values of the active force and the fraction of crossbridges in the strongly bound, weakly bound, and detached states were recorded (see Figure 3).

### 2.4. Parameter Values, Initial Conditions, and Numerical Integration Methods

All the parameter values and initial conditions for the EP model, the myofilament model, the mitochondrial energetics model, and the spatial half-sarcomere model are as described in the corresponding manuscripts and their supplementary materials: O'Hara et al. [20] for the electrophysiology model, Tran et al. [21] for the myofilament model, Cortassa et al. [23] for the mitochondrial model, and Powers et al. [24] for the half-sarcomere model. The changes made to the ORd EP model as well as the 50% reduction to the Hill coefficient in the myofilament model are detailed in Table 1. These changes were made as they align with the experimentally observed changes between control and HCM patients [16, 17]. Numerical integration of the ordinary differential equations (ODEs) was performed using the LSODA ODE differential equation solver [29].

## 3. Results and Discussion

### 3.1. Model Validation: HCM Electromechanical Single Cell

**Figure 4:**
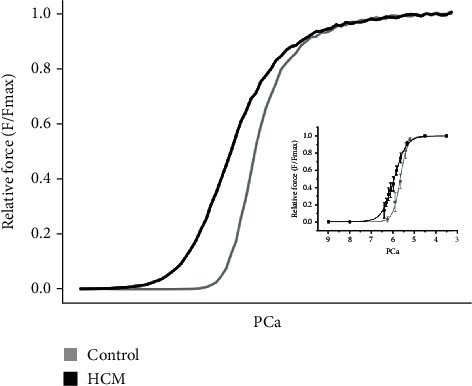
Force-pCa relationship. Simulated force-pCa relationship in control and HCM. Relative force is normalised to the maximum value that can be attained. Inset: experimental force-pCa relation of skinned HCM and control preparations obtained from human patients [17].

To validate the HCM single-cell model, we computed a steady-state force-Ca^2+^ (*F*-pCa) relationship for a sarcomere length (SL) of 2.2 *μ*m and compared the outcome with the experimental data [17]. The results are shown in Figure 4. The model reproduced the differences in total and passive forces between control and HCM, which matched the experimental data (inset).

### 3.2. Functional Electrophysiological Consequences of HCM

**Figure 5:**
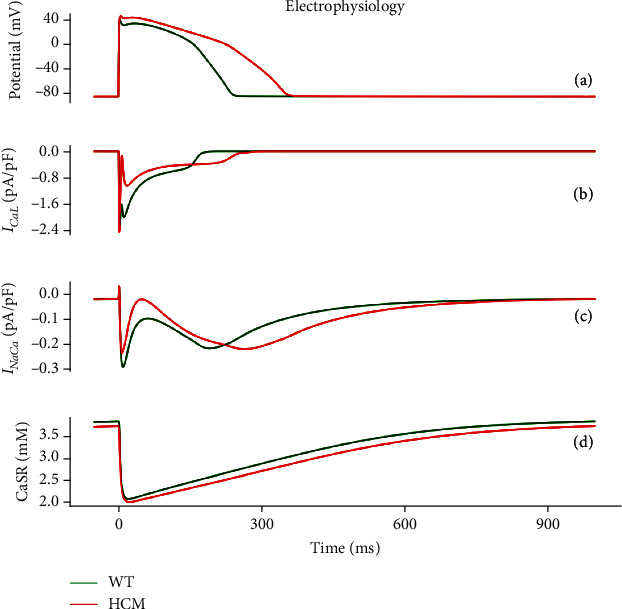
Electrophysiological properties under control (green) and HCM (red) at 1 Hz cycle length. (a) Epicardial action potential in HCM and control. (b) L-type Ca^2+^ channel current (*I*_CaL_). (c) Sodium/calcium exchange current (*I*_NaCa_). (d) Ca^2+^ release and reuptake from and to the sarcoplasmic reticulum.

**Figure 6:**
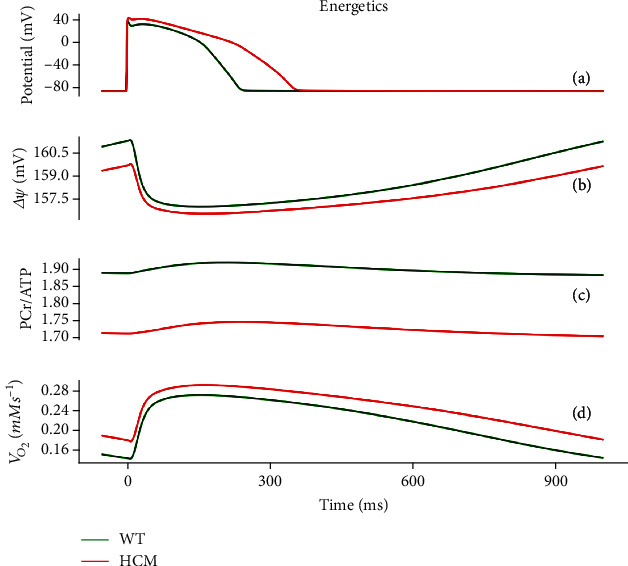
Bioenergetic properties under control (green) and HCM (red) at 1 Hz cycle length. (a) Epicardial action potentials in HCM and control. (b) Time course of change of mitochondrial membrane potential. (c) Phosphocreatine-to-ATP (PCr/ATP) ratio. (d) Oxygen consumption rate (*V*_O_2__).

Figure 5 shows the electromechanical consequences of HCM in a free-running cell from the epicardium. HCM increased the action potential duration (APD_90_) from 231 ms to 342 ms (see Figures 3(a), 5(a), and 6(a)). This is qualitatively similar to what was experimentally observed in HCM cardiomyocytes [17].

The L-type Ca^2+^ current density (*l*_CaL_) increased in HCM at the onset of AP depolarisation but reduced in amplitude during the plateau phase (see Figure 5(b)). Although the intracellular Ca^2+^ concentration [Ca^2+^]_*i*_ amplitude was similar in HCM and control (see Figure 3(b)), its kinetics was slower in HCM. The slowed kinetics of [Ca^2+^]_*i*_ (see Figure 3(b)) coupled with the transient increase in *l*_CaL_ led to an increased amplitude of the reverse mode of the Na^+^/Ca^2+^ exchanger (NCX) while leaving its forward mode unchanged (see Figure 5(c)). The initial concentration in the sarcoplasmic reticulum (SR) is lower in HCM, but in absolute terms, the release is the same as that in WT (see Figure 5(d)). The SR Ca^2+^ content is less in HCM. These factors, along with the decreased conductance of repolarising K^+^ currents (see Table 1; *G*_Kr_, *G*_Ks_, *G*_to_, and *G*_K1_), contributed to the prolongation of the action potential in HCM (see Figures 3(a), 5(a), and 6(a)).

With every heartbeat, the [Ca^2+^]_*i*_ undergoes an order of magnitude change, while the resulting force may change by three or more orders of magnitude. This biological design is believed to be motivated by the need for significant changes in developed force to efficiently pump blood.

When incomplete relaxation, or delayed relaxation, occurs (see Figure 3(d)), a residual force, and hence pressure in the ventricle, inhibits proper filling during diastole, resulting in the ejection of a smaller amount of blood during systole. In contrast, cardiac cells produce relatively small changes in [Ca^2+^]_*i*_ because ATP is required to actively decrease [Ca^2+^]_*i*_ with each heartbeat. The steep nonlinearity of the *F*-pCa relationship illustrates the small change in [Ca^2+^]_*i*_ and the large changes in force. A steep nonlinearity enables a proportionally larger change in force for a much smaller relative change in [Ca^2+^]_*i*_ per heartbeat [30, 31]. Figure 4 shows that the force-pCa relationship is left-shifted in HCM patients indicating increased Ca^2+^ sensitivity, which resulted in greater sarcomere length shortening (see Figure 3(c)) and greater developed force in HCM with a lesser magnitude of [Ca^2+^]_*i*_ (see Figure 3(d)).

The mitochondrion is a major organelle storing Ca^2+^ and regulates the [Ca^2+^]_*i*_ homeostasis with the SR [32]. The potential energy derived from the mitochondrial membrane potential (ΔΨ) is essential for the generation of ATP during oxidative phosphorylation [32, 33]. ΔΨ was reduced in HCM (see Figure 6(b)), implying less potential energy available for the generation of ATP by the enzyme ATP synthase, resulting in a potential mismatch between energy (ATP) demand and supply. This is indicated by a decreased phosphocreatine-to-ATP ratio (-9%) (see Figure 6(c)) and an increased oxygen consumption rate, or respiration rate (see Figure 6(d)). The ratio of phosphocreatine/ATP (PCr/ATP) reflects the availability of phosphocreatine and is a measure of the energy state of the myocardium [34–36]. The impaired cardiac energetics in HCM seen in our model is consistent with a multiomics profile study of 27 HCM patients and 13 controls [9], providing validation of our developed human electromechanical energetic single-cell model.

### 3.3. Sensitivity Analysis of the HCM Electromechanical Model Parameters

Table 1 shows the parameters that were modified in the control electromechanical model to obtain the HCM electromechanical model. Given the complexity of the integrated models, we performed a sensitivity analysis to identify the key parameters that have the most significant impact on the results. We therefore systematically modified each parameter in the control electromechanical model at a time to determine its effects. The results are shown and discussed below.

#### 3.3.1. Effect of the Changes on Membrane Potential

**Figure 7:**
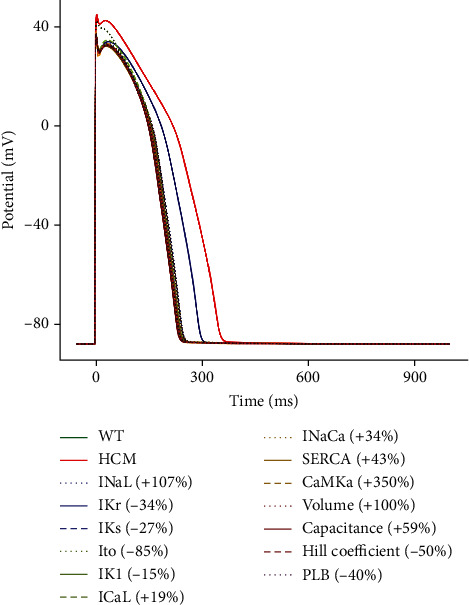
Sensitivity analysis of the model parameters compared to control and HCM at 1 Hz cycle length on the membrane potential.

The only parameter that had a significant effect on the action potential duration was the 34% reduction of *I*_Kr_ (see Figure 7). This caused the APD_90_ to increase from 231 ms to 328 ms, which is still shorter than the full HCM electromechanical model's APD_90_ of 342 ms. *I*_Kr_ is a repolarising K^+^ current that is active during the plateau phase of the action potential, and its reduction results in a delayed repolarisation or a prolonged APD.

#### 3.3.2. Effect of the Changes on CaSR

**Figure 8:**
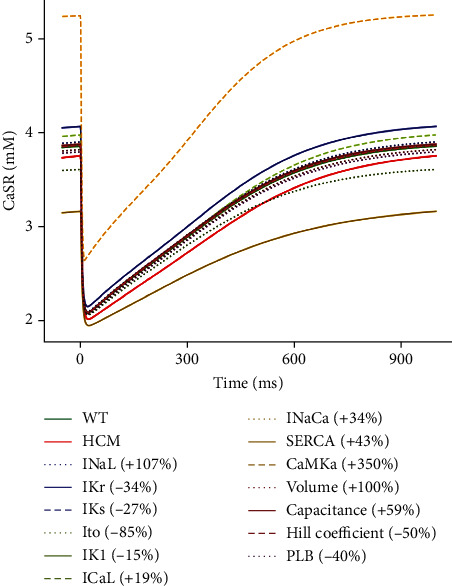
Sensitivity analysis of the model parameters compared to control and HCM at 1 Hz cycle length on *I*_CaL_.

In WT, the initial [Ca^2+^] in the SR was ~3.7 mM and had a maximal reduction to ~2.1 mM during the action potential (see Figure 8). In HCM, the initial [Ca^2+^] of the SR was ~3.6 mM with a maximal reduction to ~2 mM during the action potential (see Figure 8). All parameter changes had an effect approximately between those of WT and HCM, i.e., a maximal Ca^2+^ release of ~50% except for changes in CaMK and SERCA. CaMK had the greatest effect (see Figure 8). While the change in CaMK also had a maximal release of ~50%, the maximal [Ca^2+^] was elevated to ~5.2 mM.

In HCM, CaMK is elevated by 350%. This elevation would be expected to result in an increased SR [Ca^2+^] by stimulating the uptake of Ca^2+^ by SERCA into the SR, leading to a higher SR Ca^2+^ load. The higher SR Ca^2+^ content then provides more releasable Ca^2+^ with each subsequent action potential. This release is further enhanced by the promotion of Ca^2+^ release by direct action of CaMK on the ryanodine receptor (RyR) and the activation of CaMK by the calcium-calmodulin complex. Therefore, the higher cytosolic Ca^2+^ levels not only activate CaMK but also create a positive feedback loop. While this enhances the heart's pumping ability, it introduces a potential risk of Ca^2+^-driven arrhythmias due to the heightened release of Ca^2+^ and increased cytosolic Ca^2+^ concentrations.

#### 3.3.3. Effect of the Changes on [Ca^2+^]_*i*_

**Figure 9:**
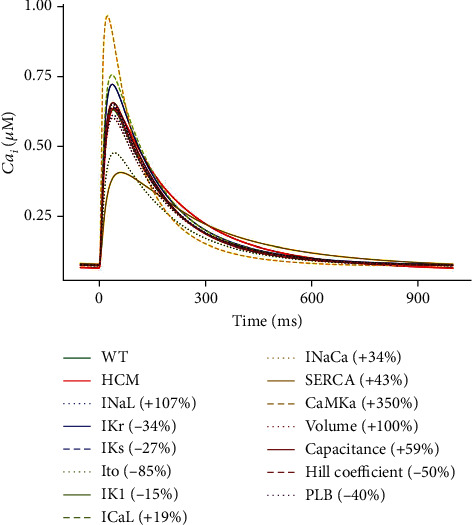
Sensitivity analysis of the model parameters compared to control and HCM at 1 Hz cycle length on [Ca^2+^]_*i*_.

The increase in CaMK had the greatest effect on the [Ca^2+^]_*i*_, increasing the amplitude from ~0.62 *μ*M to ~0.9 *μ*M (see Figure 9). Since CaMK phosphorylates and activates RyR, the channels open more easily resulting in greater SR Ca^2+^ release that will increase the amplitude of [Ca^2+^]_*i*_. Additionally, CaMK phosphorylates and inhibits phospholamban, an inhibitor of the SERCA pump. This disinhibition of the SERCA pump allows greater SR calcium reuptake by SERCA accelerating the [Ca^2+^]_*i*_ decay (see Figure 9) as Ca^2+^ is pumped back into the SR. The increased SR Ca^2+^ reuptake also replenishes the SR Ca^2+^ stores maintaining a higher SR Ca^2+^ content. This further augments the amount of releasable Ca^2+^ (see Figure 8).

*I*_CaL_ also increased the [Ca^2+^]_*i*_  amplitude from ~0.62 *μ*M to ~0.75 *μ*M. *I*_CaL_ provides the influx of trigger Ca^2+^ that initiates calcium-induced calcium release from the SR. Therefore, an increase in *I*_CaL_ would result in greater Ca^2+^ influx during the AP plateau, contributing to a higher [Ca^2+^]_*i*_ level. The increased trigger Ca^2+^ activates more RyR channels leading to greater SR Ca^2+^ release and an increase in the amplitude of [Ca^2+^]_*i*_ (see Figure 9).

The 34% increase in *I*_NaCa_ reduced the [Ca^2+^]_*i*_  amplitude from ~0.62 *μ*M to ~0.45 *μ*M (see Figure 9). *I*_NaCa_ is responsible for the extrusion of Ca^2+^ from the cardiomyocyte. Therefore, an increase in *I*_NaCa_ activity means that more Ca^2+^ is pumped out of the cardiomyocyte reducing the [Ca^2+^]_*i*_ (see Figure 9).

Most of the other parameter changes had an effect approximately intermediate between WT and HCM (see Figure 9).

#### 3.3.4. Effect of the Changes on the Active Force

**Figure 10:**
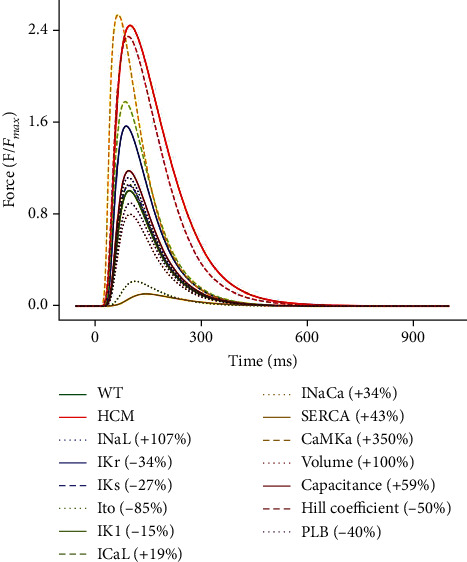
Sensitivity analysis of the model parameters compared to control and HCM at 1 Hz cycle length on the active force. IK1 is superimposed on WT.

The 350% increase of CaMK increased the active force of WT 2.5-fold (see Figure 10). CaMK phosphorylates and activates the RyR Ca^2+^ release channels, which results in greater Ca^2+^ release from the SR. The increased SR Ca^2+^ release amplifies [Ca^2+^]_*i*_, providing more Ca^2+^ to bind to troponin C and initiate myofilament crossbridge cycling. CaMK phosphorylates and inhibits phospholamban, which disinhibits SERCA. This increases SR Ca^2+^ reuptake. Faster SR Ca^2+^ reuptake allows quicker relaxation (see Figure 10) and replenishes SR Ca^2+^ stores, further increasing releasable calcium (see Figure 8). Overall, this leads to an increase in the active tension and force generation by cardiomyocytes (see Figure 10).

The 50% decrease in the Hill coefficient also increased the active force of WT ~2.3-fold (see Figure 10). The Hill coefficient quantifies the steepness of the force-Ca^2+^ relationship and quantifies the cooperative binding of Ca^2+^ to troponin C which activates the myofilaments in cardiomyocytes. With reduced steepness and consequently lower cooperativity, the binding of Ca^2+^ to troponin C occurs more gradually. Without cooperative effects, Ca^2+^ dissociates more gradually from troponin C thereby slowing relaxation and increasing active force (see Figure 10).

The 19% increase in *I*_CaL_ increased the active force of WT ~1.8-fold (see Figure 10). *I*_CaL_ provides Ca^2+^ influx during the AP plateau, acting as the trigger for calcium-induced calcium release from the SR. Increased *I*_CaL_ density would result in increased SR Ca^2+^ release. This would lead to an increase in active tension and force generation (see Figure 10). The higher Ca^2+^ level would also hasten relaxation (see Figure 10) by providing more substrate for removal, mainly by the sodium-calcium exchanger (NCX).

*I*_NaCa_ reduced the active force to 10% of WT (see Figure 10). It primarily functions by exchanging three Na^+^ into the cell for one Ca^2+^ out of the cell. An increase in *I*_NaCa_ activity (43% under HCM) leads to high Ca^2+^ extrusion, reducing the [Ca^2+^]_*i*_. By lowering [Ca^2+^]_*i*_, the increase in *I*_NaCa_ indirectly influences contractility. Reduced [Ca^2+^]_*i*_ during diastole means that there will be less Ca^2+^ available for the next contraction, leading to a decrease in contractile force (see Figure 10).

*I*_Kr_ is reduced by 34% in HCM. This reduction slows repolarisation and prolongs the APD, which lengthens the period of Ca^2+^ influx via the action of *I*_CaL_. The increased Ca^2+^ influx can enhance calcium-induced calcium release from the SR and augment [Ca^2+^]_*i*_. The higher [Ca^2+^]_*i*_ leads to increased Ca^2+^ binding to troponin C and greater myofilament crossbridge formation. This results in an increase in peak active tension and force generation and slower relaxation (see Figure 10).

The other parameter changes had a moderate effect on active force compared to HCM when all the changes were incorporated.

#### 3.3.5. Effect of the Changes on the Mitochondrial Membrane Potential (ΔΨ)

All the parameters had effects that were approximately intermediate between WT and HCM except for CaMK, SERCA, and *I*_to_ (see Figure 9). CaMK is increased by 350% in HCM and SERCA by 43%, while *I*_to_ is reduced by 85% (see Table 1).

**Figure 11:**
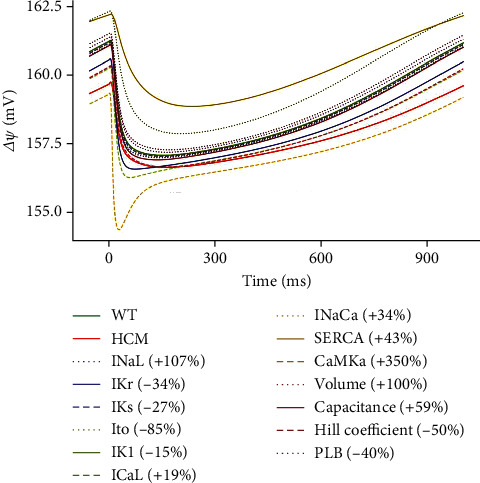
Sensitivity analysis of the model parameters compared to control and HCM at 1 Hz cycle length on mitochondrial membrane potential. IK1 is superimposed on WT.

The greatly elevated CaMK hyperphosphorylates and overactivates the RyR. This leads to excessive Ca^2+^ release from the SR and Ca^2+^ overload in the cytosol and mitochondria. Mitochondrial Ca^2+^ overload can cause the opening of the mitochondrial permeability transition pore (mPTP). The opening of the mPTP dissipates the mitochondrial membrane potential (ΔΨ) (see Figure 11) and impairs ATP production. The reduction in ΔΨ prevents normal Ca^2+^ uptake into the mitochondria, further disrupting cellular Ca^2+^ regulation. CaMK also increases metabolism and ATP demand; combining this with reduced ATP production over time depletes energy stores.

The 43% increase in SERCA had the opposite effect in that it augmented ΔΨ (see Figure 11). Increased SERCA activity accelerates Ca^2+^ reuptake into the SR during relaxation. Faster Ca^2+^ clearance from the cytosol prevents Ca^2+^ overload and reduces the entry of Ca^2+^ into the mitochondria. Since excessive mitochondrial Ca^2+^ can trigger the opening of the mPTP, the increased SERCA activity sustains ΔΨ by depolarising the inner mitochondrial membrane and preventing Ca^2+^ overload (see Figure 11). The 85% reduction of *I*_to_ in HCM also augmented ΔΨ. This was because the loss of *I*_to_ prolonged the AP and increased Ca^2+^ influx primarily through *I*_CaL_ activity. This leads to Ca^2+^ overload in the cytosol and subsequently in the mitochondria. Mitochondrial Ca^2+^ overload triggers the opening of the mPTP, and opening the mPTP dissipates ΔΨ and impairs ATP production.

#### 3.3.6. Effect of the Changes on the PCr/ATP Ratio

**Figure 12:**
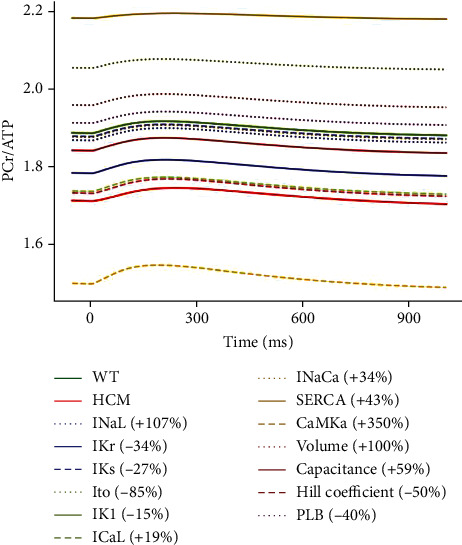
Sensitivity analysis of the model parameters compared to control and HCM at 1 Hz cycle length on the PCr/ATP ratio. IK1 is superimposed on WT.

CaMK, SERCA, and *I*_to_ had the most significant effect on the PCr/ATP ratio (see Figure 12). CaMK reduced the PCr/ATP ratio from ~1.9 in WT to ~1.5. SERCA increased the ratio from ~1.9 to ~2.2, while *I*_to_ increased it from ~1.9 to ~2.1. All the other parameters had effects that were intermediate between WT and HCM or close to WT (see Figure 12). CaMK is increased by 350% in HCM and SERCA by 43%, while *I*_to_ is reduced by 85% (see Table 1).

As a possible mechanism for CaMK, the 350% increase in CaMK activity leads to excessive Ca^2+^ release from the SR, causing cytosolic and mitochondrial Ca^2+^ overload. This may lead to increased ATP demand to support enhanced contractile activity and ion transport. This higher ATP demand then leads to the utilisation of PCr stores to regenerate ATP and consequently a reduction in the PCr/ATP ratio (see Figure 12).

The 85% reduction in *I*_to_ leads to a prolongation of the APD, increasing Ca^2+^ influx through *I*_CaL_. This causes Ca^2+^ overload thereby activating SERCA, which consumes ATP. The resulting increase in ATP consumption leads to an increase in the PCr/ATP ratio. An additional mechanism is that excess mitochondrial Ca^2+^ triggers sustained the opening of the mPTP, which depolarises the inner mitochondrial membrane and impairs ATP production.

The 43% increase in SERCA activity means that more Ca^2+^ is pumped back into the SR. This can reduce [Ca^2+^]_*i*_, implying that less energy is required to maintain ion gradients, such as NCX activity. With reduced energy expenditure on calcium handling, more ATP is available for the regeneration of PCr. These effects can ultimately contribute to the increased PCr-to-ATP ratio (see Figure 12).

#### 3.3.7. Effect of the Changes on the Oxygen Consumption Rate (*V*_O_2__)

**Figure 13:**
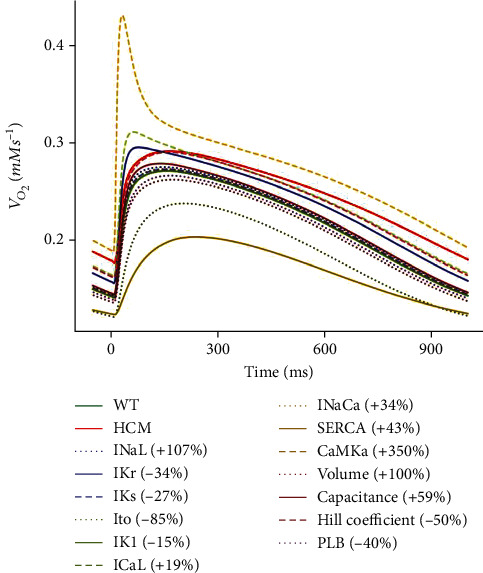
Sensitivity analysis of the model parameters compared to control and HCM at 1 Hz cycle length on the oxygen consumption rate (*V*_O_2__). IK1 is superimposed on WT.

CaMK, SERCA, and *I*_to_ had the most significant effects on *V*_O_2__ (see Figure 13). CaMK had a maximum peak value of 0.43 mM∙s^−1^, *I*_to_ had a maximum amplitude of 0.24 mM∙s^−1^, and SERCA's maximum amplitude was 0.21 mM∙s^−1^. The maximum amplitude for WT was 0.27 mM∙s^−1^. The other parameters had maximum amplitudes that were comparable with WT or intermediate between WT and HCM (see Figure 13).

### 3.4. Functional Consequences of HCM on Crossbridge Cycling

Using the spatial multifilament half-sarcomere model [24–27], we further investigated the functional consequences of HCM on crossbridge cycling. The half-sarcomere model is a three-state model with states for crossbridge attachment, force generation, and detachment. The transitions between these states determine both the forces experienced by each crossbridge and the rate of ATP utilisation associated with the number of times each crossbridge detaches from the thin filament and hydrolyses a subsequent ATP for subsequent binding and force generation. There is no thin filament regulation in the model as Ca^2+^ regulation is not considered. So, we could not couple it to an electrophysiological model as was done in the electromechanical model in Sections 2.1 and 3.2. Instead, Ca^2+^ activation/regulation is considered indirectly in the model through actin permissiveness, which can be set to values between 0 (nonpermissive) and 1 (fully permissive). This implies that, in this setting, troponin/tropomyosin interactions are combined into a single variable. While this simplification allows for meaningful qualitative observations, it may not fully capture the complexity of actual molecular mechanisms.

We used as input to the model the experimental [Ca^2+^]_*i*_ transient profiles of control and HCM patients (see Figure 2). These were digitised, and each timepoint of the pertinent profile was run until reaching the steady state, after which the steady-state value was recorded. This was done throughout the time course of each [Ca^2+^]_*i*_ transient profile. In each case, the minimum value of the [Ca^2+^]_*i*_ transient in the control condition was mapped to an actin permissiveness of 0.2 and the maximum [Ca^2+^]_*i*_ transient value was mapped to an actin permissiveness of 1. [Ca^2+^]_*i*_ transient values were mapped proportionally between these actin permissiveness values.

**Figure 14:**
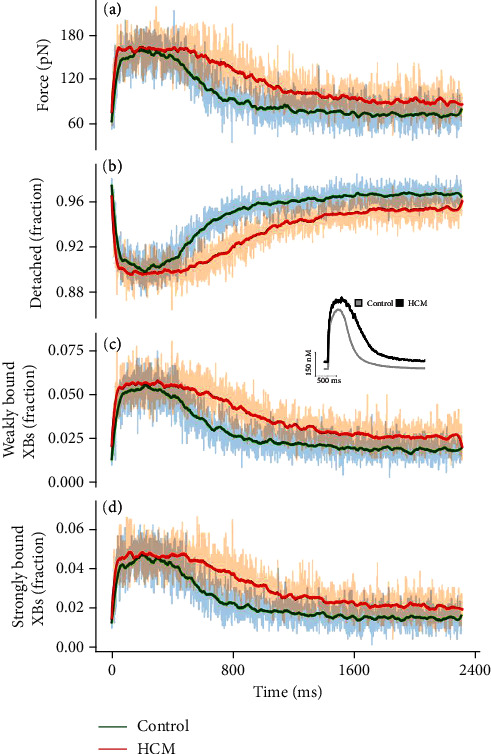
Crossbridge states in control and HCM with the spatial multifilament half-sarcomere model. (a) The developed force in control (green) and HCM (red). (b) The fraction of crossbridges in the detached state during the time course of the [Ca^2+^]_*i*_ transient profile. (c) The fraction of crossbridges in the weakly bound state during the time course of the [Ca^2+^]_*i*_ transient profile. (d) The fraction of crossbridges in the strongly bound state during the time course of the [Ca^2+^]_*i*_ transient profile.

Figure 14(a) shows the active force in control and HCM for the [Ca^2+^]_*i*_ transient profile (inset). The force in both conditions follows closely the input [Ca^2+^]_*i*_ transient profile. This should not be the case; that is, the force should not depend exactly on the [Ca^2+^]_*i*_ transient profile as experimental transients contain artifacts from a nonlinear response of the Ca^2+^-sensing dyes. The [Ca^2+^]_*i*_ transient and the force should be dynamically generated from a dynamic free-running cell (model) based on the interplay of ion channels and other cellular components.

Unfortunately, as discussed above, the half-sarcomere model does not consider Ca^2+^ regulation directly but only through actin permissiveness. Thus, we were unable to couple the half-sarcomere model to an electrophysiology model. This has certain implications that merit consideration. The lack of direct Ca^2+^ regulation may affect the dynamic interplay between ion channels and cellular components, which could affect how well our model mimics the complexities of a real cell. Additionally, we acknowledge that experimental [Ca^2+^]_*i*_ transient profiles inherently contain artifacts, and our model, by not directly incorporating Ca^2+^ regulation, may not fully capture the dynamic responses expected in a free-running cellular environment. Nevertheless, the results are qualitatively sound.

Figures 14(b)–14(d) show that on average, in the rest state, the control condition has ~97.5% of crossbridges in the detached state (HCM ~96%), ~1.5% in the weakly bound state (HCM ~2.4%), and~1.5% in the strongly bound state (HCM ~1.6%).

In the weakly bound state, the myosin head has broken ATP into ADP (adenosine diphosphate) and phosphate (Pi). During this phase, the head has just attached to actin but has not yet released ADP or Pi. In this state, the myosin is only partially bound to actin and little or no force is produced. The myosin head then rotates into a strongly bound configuration, while ADP and Pi are released. In this state, myosin performs the primary force-producing “power stroke” of the crossbridge cycle as it pulls on the actin filament [37]. This implies that even at rest, HCM cardiomyocytes are more primed to generate force.

Throughout the time course of the active force profiles (see Figure 14(a)), the HCM condition has more crossbridges in the weakly bound and strongly bound states. At about 1000 ms, the control condition has ~96% detached crossbridges, while this is ~94% in HCM (see Figure 14(b)). For the weakly bound state, it is ~2.5% crossbridges for controls and 3.75% for HCM (see Figure 14(c)), and for the strongly bound state, it is ~2% and ~3%, respectively (see Figure 14(d)). This proportion is maintained throughout the rest of the time course of the profiles. This shows that from heartbeat to heartbeat, in the HCM condition, more crossbridges are in the weakly bound (very low force generating) and in the strongly bound (force generating) states compared to the control condition.

Figure S1 in the supplementary material shows the result from a second [Ca^2+^]_*i*_ transient profile from HCM patients with a MYBPC3 mutation. It shows a similar trend to the results in Figure 14 and corroborates that HCM indeed has a higher proportion of crossbridges in the force-generating states both during contraction and in the relaxed state, compared to control conditions.

### 3.5. Implications and Significance of Major Findings

In this study, we developed a human ventricular electromechanical model that incorporated experimentally observed electrophysiological and mechanical changes during HCM. We also used a half-sarcomere to investigate the effects of reported HCM Ca^2+^ transients to investigate the mechanisms underlying altered crossbridge cycling in HCM. Our simulations implicated CaMK, SERCA, and *I*_to_ as key contributors to HCM pathophysiology. In HCM, CaMK is elevated by 350%, SERCA is increased by 43%, and *I*_to_ is reduced by 85%. Identifying elevated CaMK, elevated SERCA, and reduced *I*_to_ as major players in HCM pathophysiology provides crucial insights into the underlying molecular and cellular mechanisms of the disease. An CaMK increase by itself leads to excessive Ca^2+^ release from the SR and Ca^2+^ overload in the cytosol and mitochondria. While a compensatory elevated SERCA activity enhances Ca^2+^ reuptake into the SR, it also provides more Ca^2+^ for SR release during subsequent beats, helping maintain contractility. However, the 85% decrease in *I*_to_ serves to prolong the APD and provides more time for Ca^2+^ influx into the cytosol. This continuous cycle is at the cost of greater energy demand beyond supply.

Consequently, strategies to reduce CaMK and SERCA and increase *I*_to_ activity or modulate their downstream effects can potentially offer new treatment options for individuals with HCM. In addition, elevated CaMK levels in tandem with increased SERCA activity and reduced *I*_to_ levels could potentially help identify individuals with HCM at higher risk or with more severe disease and guide clinical management decisions.

### 3.6. Relevance to Previous Studies

Liu et al. [18] developed a computational model to investigate the behaviour of electromechanical coupling in the human left ventricular cardiomyocyte and explore the roles of various factors in the pathological manifestations of HCM cardiomyocyte and their changes in response to drug treatment. Their study revealed that in addition to the characteristic changes in *I*_CaL_ and intracellular Na^+^ concentration, almost all other ion channel current densities and intracellular ion and molecule concentrations suffered from marked changes. This agrees with our model of HCM based on the experimental changes observed by Coppini et al. [16, 17] (see Table 1).

Pioner et al. [19] used a computational model to test the mechanism responsible for a preserved twitch duration with the underlying prolonged AP and Ca2^+^ transients in a HCM-linked MYBPC3 mutation. They found that electrophysiological changes (prolonged action potentials and Ca^2+^ transients) appear to counterbalance the faster crossbridge cycling, ultimately preserving the amplitude and duration of cardiac contraction, at the expense of cardiac electrical stability and diastolic function. This supports our result of the spatial half-sarcomere model showing more crossbridges in the bound state in HCM (see Figure 14).

Forouzandehmehr et al. [38] developed a metabolite-sensitive computational model of human induced pluripotent stem cell-derived cardiomyocyte (hiPSC-CM) electromechanics to investigate the pathology of R403Q HCM mutation and the effect of mavacamten (MAVA), blebbistatin (BLEB), and omecamtiv mecarbil (OM) on the cell mechanoenergetics. Their model captured the prolonged contractile relaxation due to the R403Q mutation without assuming any further modifications such as an additional Ca^2+^ flux to the thin filaments. However, their model did not employ a spatial model and did not consider other electrophysiological changes to the HCM cardiomyocyte.

### 3.7. Limitations of These Simulations

The limitations of the ORd model [20], the Tran et al. model [21, 22], and the mitochondrial model [23] have been discussed in the respective model papers. Our single-cell simulations share these limitations.

In addition to these limitations, there is no thin filament regulation in the spatial myofilament model as Ca^2+^ regulation is not considered. Therefore, we could not couple it to an electrophysiological model as was done with the single-cell electromechanical model in Sections 2.1 and 3.2. Instead, Ca^2+^ activation/regulation is considered indirectly in the model through actin permissiveness, which can be set to values between 0 (nonpermissive) and 1 (fully permissive). This implies that, in this setting, troponin/tropomyosin interactions are combined into a single variable.

While this simplification allows for meaningful qualitative observations, it may not fully capture the complexity of the actual cellular mechanisms. This has potential implications for the overall accuracy and biological significance of our model. The absence of direct Ca^2+^ regulation, a fundamental aspect of muscle contraction, may influence the dynamic interplay between ion channels and cellular components. This could impact how well the model mimics the intricacies of a living cell.

We also used as input to the spatial half-sarcomere model prerecorded [Ca^2+^]_*i*_ transients. Hence, the force output depends exactly on the [Ca^2+^]_*i*_ transient profile. This should not be the case as experimental transients contain artifacts from a nonlinear response of the Ca^2+^-sensing dyes. The [Ca^2+^]_*i*_ transient and the force should be dynamically generated from a dynamic free-running cell (model) based on the interplay of ion channels and other cellular components. Unfortunately, as discussed above, the half-sarcomere model does not consider Ca^2+^ regulation directly but only through actin permissiveness. Thus, we were unable to couple the half-sarcomere model to an electrophysiology model.

It may have also been beneficial to use a greater number of samples for the prerecorded [Ca^2+^]_*i*_ transients, but more samples which compared control with HCM were difficult to source. Ultimately, even if more samples were used, the results would still be subject to the problem alluded to in the above paragraph, i.e., the force output depending exactly on the [Ca^2+^]_*i*_ transient profile. Consequently, more targeted experiments and experimental data focused solely on this goal should be performed and collected.

At present, the modifications made to the EP and myofilament models to reflect the electromechanical profile of HCM cardiomyocytes are based on the experimental data from Coppini et al. [16, 17]. It would be beneficial to have a more comprehensive validation of the modified model against additional experimental data to ensure its accuracy in capturing HCM characteristics.

While it is important that potential limitations of the models used in this study are made explicit, these do not fundamentally affect the conclusions that can be drawn on likely mechanisms by which HCM alters electrophysiological activity, contractility, energy metabolism regulation, and crossbridge cycling.

## 4. Conclusions

HCM cardiomyocytes exhibited greater contractility (Figure 5) due to the combination of (a) slowed [Ca^2+^]_*i*_ decay kinetics, (b) reduced SERCA activity and phospholamban (PLB), (c) greater myofilament Ca^2+^ sensitivity, and (d) a higher proportion of crossbridges in force-generating states compared to the control condition. All of these combined to produce, or were derived from, Ca^2+^ accumulation in the cytoplasm. This greater cardiomyocyte contractility is what enables the heart, and in particular, the left ventricle, to maintain a relatively normal ejection fraction despite the wall hypertrophy in HCM. Our results show that a relatively normal function is maintained in HCM due to Ca^2+^ handling abnormalities, but to achieve normal or close to normal function, the cardiomyocytes have a greater maximal oxygen consumption rate. Our results also implicate significantly elevated CaMK, increased SERCA activity, and significantly reduced *I*_to_ as the key contributors to HCM pathophysiology. These combine to elevate the [Ca^2+^]_*i*_ and lead to increased ATP consumption to maintain contractility. CaMK, SERCA, and *I*_to_ thus serve as potential therapeutic targets and diagnostic or prognostic markers for HCM.

## Figures and Tables

**Table 1:** Hypertrophic cardiomyopathy (HCM) functional changes [16, 17] made to the electromechanical single-cell model.

Parameter	HCM
Volume	+100%
Capacitance	+59%
*G*_NaL_	+107%
*G*_Kr_	-34%
*G*_Ks_	-27%
*G*_to_	-85%
*G*_K1_	-15%
*G*_CaL_	+19%
*G*_NaCa_	+34%
SERCA	-43%
CaMKa	+350%
PLB	-40%
Hill coefficient^∗^	-50%

^∗^Change made to the myofilament model of the coupled single model. *G*_NaL_ is the maximum conductance of the late sodium current, *G*_Kr_ is the maximum conductance of the rapid delayed rectifier K^+^ current, *G*_Ks_ is the maximum conductance of the slow delayed rectifier K^+^ current, *G*_Kr_ is the maximum conductance of the rapid delayed rectifier K^+^ current, *G*_to_ is the maximum conductance of the transient outward K^+^ current, *G*_K1_ is the maximum conductance of the inward rectifier K^+^ current, *G*_CaL_ is the maximum conductance of the Ca^2+^ current through the L-type Ca^2+^ channel, *G*_NaCa_ is the maximum conductance of the Na^+^/K^+^ exchange current, SERCA is the sarco/endoplasmic reticulum Ca^2+^-ATPase pump, CaMKa is the fraction of active Ca^2+^/calmodulin-dependent protein kinase II binding sites, PLB is phospholamban, and the Hill coefficient is a measure of the Ca^2+^ sensitivity of the cardiomyocyte.

## Data Availability

The data and source code are available by contacting the corresponding author upon request.

## Supplementary Materials

Supplementary Materials The figure shows the crossbridge states resulting from a second [Ca^2+^]_*i*_ transient profile from HCM patients with a MYBPC3 mutation using the spatial multifilament half-sarcomere model.
