# Supplementary material for: An In Silico Cardiomyocyte Reveals the Impact of Changes in CaMKII Signalling on Cardiomyocyte Contraction Kinetics in Hypertrophic Cardiomyopathy
Source: Biomed Res Int. 2024 Mar 25;2024:6160554. doi: 10.1155/2024/6160554 (PMC10985279; doi:10.1155/2024/6160554)
Supplement: Supplementary Materials — The figure shows the crossbridge states resulting from a second [Ca2+]i transient profile from HCM patients with a MYBPC3 mutation using the spatial multifilament half-sarcomere model. [file 6160554.f1.docx]

## Supplementary Materials


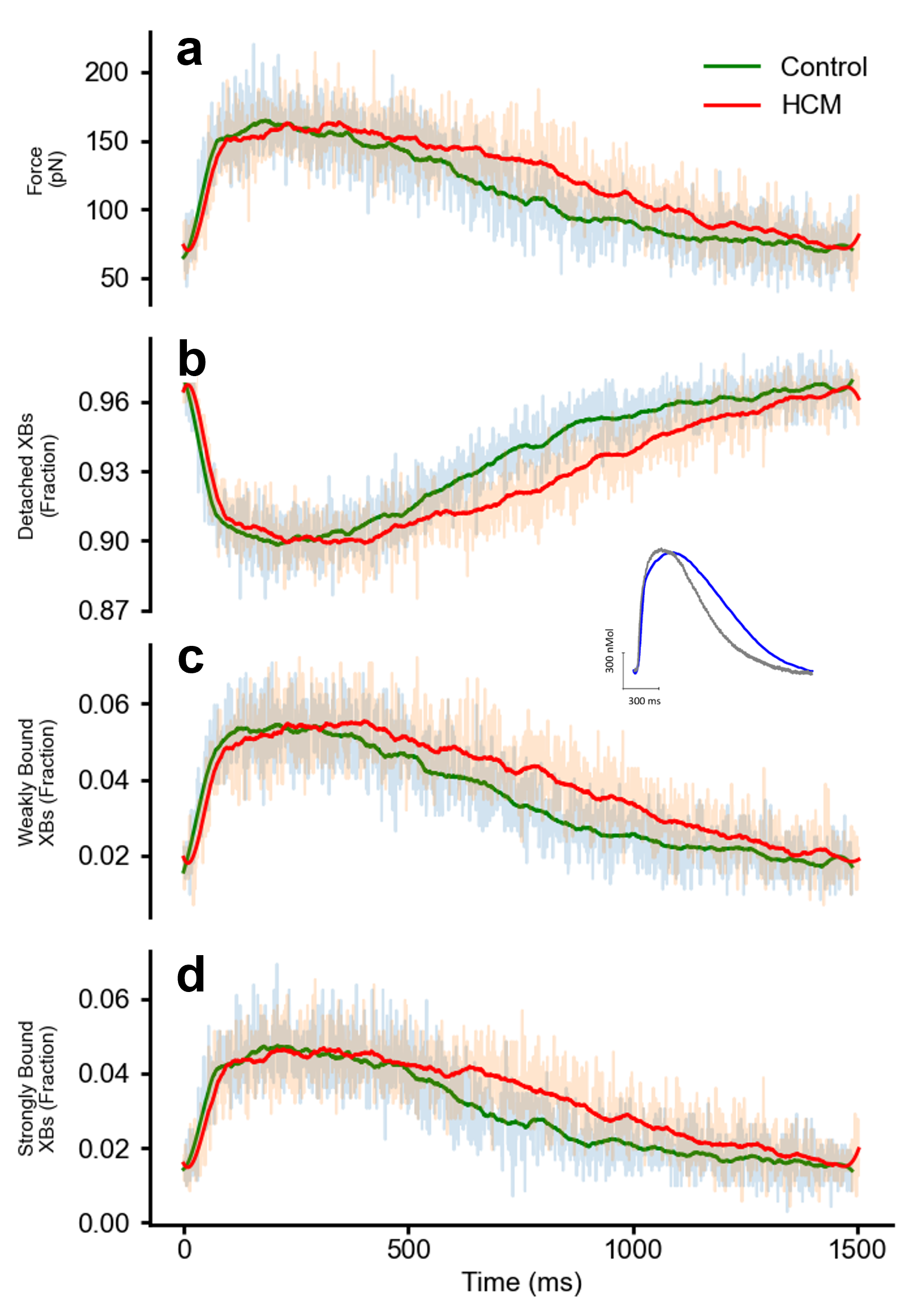


Figure S1: Crossbridge states in control and HCM (MYBPC3 mutations) with the spatial multifilament, half sarcomere model. (a) The developed force in control (green) and HCM (red). (b) The fraction of crossbridges in the detached state during the time course of the $\left[ {Ca}^{2+} \right]_{i}$ transient profile. (c) The fraction of crossbridges in the weakly bound state during the time course of the $\left[ {Ca}^{2+} \right]_{i}$ transient profile. (d) The fraction of crossbridges in the strongly bound state during the time course of the $\left[ {Ca}^{2+} \right]_{i}$ transient profile.
